# Molecular Dynamics Insights into Peptide-Based Tetrodotoxin Delivery Nanostructures

**DOI:** 10.3390/molecules30010061

**Published:** 2024-12-27

**Authors:** Shenghan Song, Xinyu Xia, Temair Shorty, Tongtong Li, Amy O. Stevens, Chao Zhao, Yi He

**Affiliations:** 1 Department of Chemistry & Chemical Biology, The University of New Mexico, Albuquerque, NM 87131, USA; 2 Department of Chemical and Biological Engineering, University of Alabama, Tuscaloosa, AL 35487, USA; 3 Center for Convergent Biosciences and Medicine, University of Alabama, Tuscaloosa, AL 35487, USA; 4 University of New Mexico Comprehensive Cancer Center, The University of New Mexico, Albuquerque, NM 87131, USA; 5 Translational Informatics Division, Department of Internal Medicine, The University of New Mexico, Albuquerque, NM 87131, USA

**Keywords:** tetrodotoxin (TTX), Site-1 sodium channel blocker (S1SCB), local anesthetics, peptide-based nanostructures, sustained-release drug delivery, molecular dynamics simulations, TTX encapsulation, nanoparticle stability

## Abstract

Tetrodotoxin (TTX), a potent Site-1 sodium channel blocker (S1SCB), offers highly effective local anesthetic properties with minimal addiction potential. To fully leverage TTX’s capabilities as a local anesthetic, it is crucial to develop a drug delivery system that balances its systemic toxicity with its therapeutic efficacy. Recent studies have shown that peptide mixtures, derived from fragments of Site-1 sodium channel proteins and enhanced with hydrophobic tails (designated MP1 and MP2), can self-assemble into nanostructures that exhibit remarkable sustained-release capabilities for TTX. Despite the profound impact that the addition of a hydrophobic tail has on altering the release behavior of the original peptides, the atomic-level interactions and mechanisms underlying this phenomenon remain poorly understood. In this study, a combination of ColabFold and molecular dynamics (MD) simulations were used to investigate the binding interactions between TTX and the nanostructures formed by MP1 and MP2 at an atomic level. Our findings agree with experimental observations and indicate that the MP1/MP2 nanostructure demonstrates greater stability and higher binding affinity for TTX compared to their non-modified counterparts, P1 and P2. The analysis of the simulations revealed that charged amino acids, specifically aspartic acid (ASP) and glutamic acid (GLU), on the peptides are crucial for strong TTX binding and serve as the primary functional sites. Additionally, the stability of the nanostructure significantly affects TTX binding affinity, elucidating why P1, P2, MP1, and MP2 exhibit different binding capabilities despite containing identical charged residues. The results reported here may provide fundamental information to drive future research and enhance the development of TTX-based drug delivery systems.

## 1. Introduction

The development of drug–carrier systems for prolonged local anesthesia offers significant potential for effective pain relief while reducing dependence on opioid analgesics. Both traditional amino-amide and amino-ester local anesthetics, such as lidocaine and bupivacaine, and emerging Site-1 sodium channel blockers (S1SCBs), such as tetrodotoxin (TTX) and saxitoxin (STX), exhibit strong local analgesic effects. However, conventional local anesthetics are associated with severe neurotoxicity and myotoxicity, and their duration of action is often insufficient [1,2,3,4,5,6]. Although less addictive than opioids, the prolonged use of these agents can lead to irreversible toxic effects. In contrast, S1SCBs such as TTX and STX do not exhibit local neurotoxicity or myotoxicity. Prior studies suggest that combining TTX with conventional local anesthetics can enhance analgesia’s duration and efficacy [7]. However, TTX binds to and inhibits sodium channel proteins such as NaV_1.7_ and NaV_1.8_ [8], contributing to its systemic toxicity, which significantly limits their therapeutic and clinical application [7,9]. Therefore, developing an efficient delivery system that can deliver S1SCBs to specific sites to prevent systemic toxicity is a critical research priority, particularly with a focus on enhancing the loading capacity and stability of peptide-based nanostructures [10,11].

Current TTX sustained-release delivery systems include photo-triggerable liposome-based systems, polymersomes/hydrogels, polymer–TTX conjugates and ultrasound-triggered local anaesthesia [12]. Rwei and colleagues, along with Zhan and others, designed photo-triggerable liposome systems that release encapsulated TTX through light-induced peroxidation reactions or temperature-induced phase transitions [13,14,15,16]. These systems offer advantages such as adjustable, on-demand release with high encapsulation efficiency. However, they also present challenges for clinical use, including the need for specific laser frequencies, which complicate the balance between laser damage and penetration depth. Additionally, careful control over TTX dosage during repeated release cycles is required to achieve effective local anesthesia without systemic toxicity. These limitations hinder the broader clinical application of photo-triggerable liposomes as a TTX delivery system. Li et al. developed emulsion-induced polymersomes and hydration-induced void-containing hydrogels capable of physically encapsulating TTX to achieve sustained release simply and effectively [17]. However, liposomes, polymersomes, and hydrogels share a similar limitation: a lack of precise dose control for TTX. This results in an initial burst release of TTX, which restricts the maximum injectable dose. Following the burst, the TTX release rate gradually decreases, often leading to the release of TTX at concentrations that are no longer therapeutically effective. Zhao et al. [18] used poly(triol dicarboxylic acid)-co-poly(ethylene glycol) (TDP) conjugated with TTX as a carrier to design and synthesize prolonged duration local anesthesia with minimal toxicity. With polyethylene glycol (PEG) acting as a chemical permeation enhancer (CPE), the TTX polymer conjugated system achieved stable binding of TTX and extended local anesthetic effects lasting several days. The polymer and PEG molecules are biodegradable, and the formulation can be stored at room temperature as a solid, offering excellent potential for clinical use. However, challenges remain in simplifying the formulation preparation methods and addressing potential local and systemic toxicity from PEG, TDP, and their metabolites.

Recently, to leverage the specificity of TTX for sodium channels, Ji et al. [19] developed a peptide-based nanostructure delivery system using short peptides P1 (sequence: TQDYWEN) and P2 (sequence: CGEWIET), derived from the TTX-binding site of Site-1 sodium channel proteins and containing critical amino acids for TTX binding such as aspartic acid (D) and glutamic acid (E). To promote self-assembly into nanostructures, modifications to these peptides were carried out by adding hydrophobic amino acid tails (sequence: FFFLL-G-) to their N termini, resulting in modified peptides MP1 and MP2, respectively. This hydrophobic modification enhances interactions among peptide molecules, facilitating their assembly into nanostructures capable of sustained TTX release. Notably, nanostructures formed with MP1 and MP2 demonstrated superior sustained-release capacity for TTX compared to those formed with alkane tails. Peptide-based carriers offer key advantages over polymer-based systems, particularly in terms of biocompatibility, and experimental results indicated that nanofibers composed of self-assembled MP1 and MP2 could encapsulate TTX and release it over 12 h.

While these experimental findings underscore the potential of peptide-based TTX delivery systems, it is still unclear why adding specific hydrophobic tails to the original P1 and P2 would lead to the formation of a nanostructure and slow down the release of TTX. The molecular interactions, such as key TTX-binding residues and peptide–peptide interactions, are crucial for designing more effective delivery systems. All-atom molecular dynamics (MD) simulations have been proven to be a powerful tool for probing the protein-protein and protein-ligand interactions [20,21,22,23]. Doll et al. designed a series of self-assembled nanostructures based on peptide chains and conducted experiments to prove the feasibility of forming nanostructures [24,25]. Recent advances in computing power and innovations such as AlphaFold [26] and ColabFold [27], which allow for rapid peptide structure prediction, have opened new avenues to bolster this research.

Here, a combination of computational chemistry approaches, specifically all-atom MD simulations in combination with ColabFold, were used to explore critical insights into the interaction mechanism of TTX-sustained-release carriers observed in prior experiments by Ji et al. [19]. We generated initial peptide structures using ColabFold and constructed MD simulation systems with TTX and peptide nanostructures. We aimed to investigate the interaction patterns of nanostructures composed of P1 and P2, or MP1 and MP2, and sought to provide information on the molecular mechanisms underlying their experimentally observed sustained-release effects. Our analysis identified the binding interactions between key amino acids and TTX, and suggested that peptide local structure stability may be a key factor in forming nanostructures. Our results not only agree with the experimental findings of Ji et al., [19] but also provide information at atomic resolution. By offering a molecular-level understanding of the interactions driving TTX encapsulation and release, such information can facilitate the development of more efficient and biocompatible TTX delivery systems.

## 2. Results

### 2.1. Effect of a Secondary Structure of Peptide Sequence on Self-Assembly and TTX-Sustained-Release Ability

**Figure 1:**
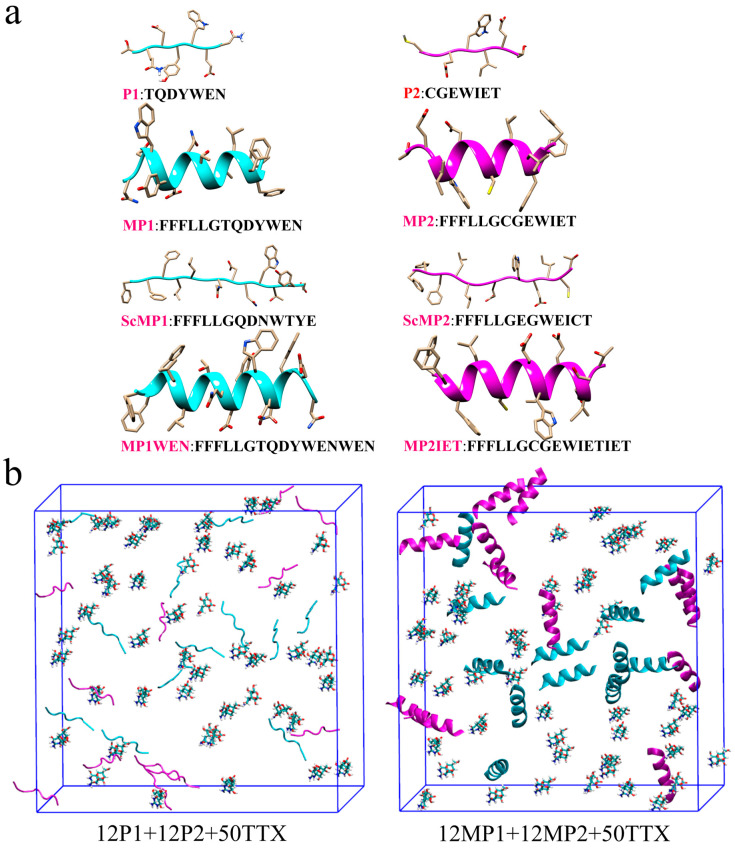
(**a**) Predicted initial structures of the peptide chains generated using Colabfold2. (**b**) Initial structures of simulation boxes for P1+P2+TTX and MP1+MP2+TTX systems. Water molecules were hidden. All the molecules were randomly filled into boxes.

As can be seen in Figure 1a, Colabfold suggests that the secondary structures of P1 and P2 are random coils, while the secondary structures of MP1 and MP2 are α-helices. As shown in Figures S1 and S2, for each Colabfold peptide prediction, their per-residue Local-Distance Difference Test (pLDDT) scores are above 70. pLDDT scores above 70 indicate that the predicted structures are reliable as suggested in the original AlphaFold 2 paper [26] and AlphaFold Protein DataBank [28]. Although predictions using Colabfold cannot be used as a rigorous basis for evaluation, secondary structure prediction results can still highlight the differences between P1/P2 and MP1/MP2. These results suggest that lacking a stable secondary structure in P1 and P2 may affect the stability of their self-assembled nanostructure as observed in previous work on peptides [29]. This also explains why Ji et al. reported that MP1 and MP2 can self-assemble to form nanofibers [19]. To ensure the formation of nanostructure is not a result of using longer peptide chains, the initial structures of the peptide sequences scrambled MP1 (ScMP1) and scrambled MP2 (ScMP2) from the experiments of Ji et al. were predicted using Colabfold. It is clear that ScMP1 and ScMP2 have the same amino acid composition and sequence length as MP1 and MP2, but their secondary structures are random coils, similar to P1 and P2. As reported in prior experiments, although ScMP1 and ScMP2 can self-assemble into nanofibers, they show a limited ability to sustain the release of TTX.

**Figure 2:**
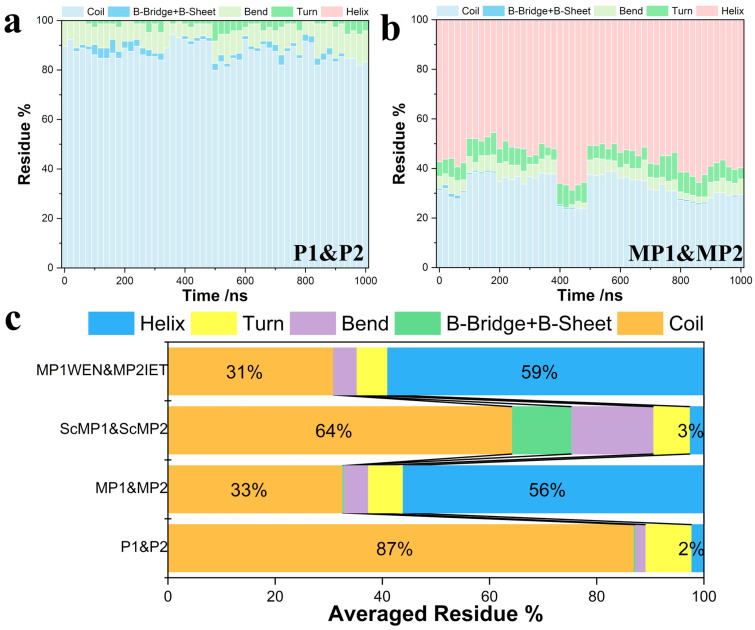
Secondary structure analysis of peptides. All the results are the average of 10 trajectories. (**a**) The proportion of secondary structures of peptide chains P1 and P2 during the simulation time. (**b**) The proportion of secondary structures of peptide chains MP1 and MP2 during the simulation time. (**c**) The average value of the proportions of various secondary structures throughout the simulation.

As a separate evaluation to cross-validate with ColabFold predictions, multiple MD simulations were used to probe the second structure preferences and interaction patterns between TTX and peptides, as shown in Figure 2. Figure 2a,b show the statistic results of the secondary structure analysis of the last 100 ns of the 10 separate trajectories for each of the P1&P2 and MP1&MP2 systems (system 1 and system 2 in Table 1), respectively. As can be seen from Figure 2, the percentage of helices in P1&P2 trajectories is close to 0%, while the proportion of helices in MP1&MP2 is close to 60% in all simulation trajectories. To further compare the secondary structure of representative peptide chains (P1&P2, MP1&MP2, ScMP1&ScMP2), the average proportion of secondary structures was calculated and displayed in Figure 2c. In the ScMP1&ScMP2 system, there is a higher percentage of regular secondary structures as compared to the P1&P2 system, which may explain its capability of forming nanostructures. These results illustrate that secondary structure analysis based on molecular dynamics simulations yields results consistent with ColabFold and prior experimental observations.

It is clear that these two different methods (MD simulation and Colabfold prediction) gave similar results for the secondary structure of the peptide chain, which suggests stable secondary structures of the peptide sequences play an important role in the nanostructure’s self-assembly and TTX-sustained-release capabilities.

### 2.2. The Binding Ability of GLU and ASP as Key Amino Acids to TTX

**Figure 3:**
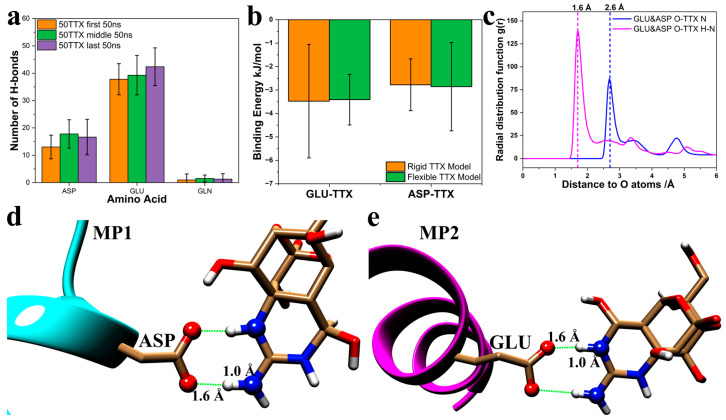
Analysis of the interaction patterns between ASP, GLU, and GLN. (**a**) Number of hydrogen bonds between ASP/GLU/GLN and TTX for three time periods of the simulation. The results are an average of ten trajectories. First 50 ns: 0–50 ns. Middle 50 ns: 500–550 ns. Last 50 ns: 950–1000 ns. (**b**) The binding energy between ASP/GLU and TTX. (**c**) The radial distribution functions of TTX to ASP&GLU. The reference atoms were oxygen atoms on residues ASP and GLU. The pink curve shows the distribution of N atoms (on TTX) around the referenced O atoms, while the blue curve shows the distribution of H atoms (Connected to N atoms on TTX) around referenced O atoms. (**d**) The binding pattern between ASP and TTX. (**e**) The binding pattern between GLU and TTX. Oxygen atoms are shown in red, nitrogen atoms in blue, and hydrogen atoms in white. Green lines represent hydrogen bonds.

Previous research on sodium ion channel proteins has shown that several hydrophilic amino acids, particularly GLU and ASP, are located at the entrance to the TTX and STX binding sites [30,31,32,33,34]. The binding of TTX and STX causes the sodium ion channel protein to lose its ability to deform and change the pore size, thereby hindering the channel protein’s normal function of controlling the entry and exit of sodium ions. In the experiments of Ji et al. [19], mutating GLU (E) to GLN (Q) reduces the nanostructure’s ability to sustain-release TTX. This result likely occurs because the binding ability of GLU and ASP to TTX is significantly stronger than that of GLN. Based on the prediction results of Colabfold (Figure S1), the secondary structure of MP1 and MP2 in which one GLU was mutated into GLN did not change significantly and remained as a helix. As reported in prior experiments and current theoretical predictions, it is likely that the ability of MP1&MP2 with an E-to-Q mutation to self-assemble into a nanostructure is not significantly affected, but the reduction in the number of GLU residues would weaken the binding to TTX. To prove this, the number of hydrogen bonds and binding energies of ASP, GLU and GLN with TTX were analyzed and calculated, as shown in Figure 3a,b. Figure 3a shows the average number of hydrogen bonds in ten simulation trajectories in three time periods: first 50 ns (0–50 ns), middle 50 ns (500–550 ns) and last 50 ns (950–1000 ns). In terms of the number of hydrogen bonds, the ability of ASP and GLU to bind TTX is dozens of times stronger than that of GLN. This significant gap in the ability of amino acids to bind TTX is very consistent with experimental observations that the E-to-Q mutation weakens the peptide’s ability to bind and slowly release TTX. Moreover, there is a small increase in the number of hydrogen bonds in simulation trajectories during three different periods. This trend implies that as the MD simulation proceeds, the binding configuration between peptides and TTX is continuously optimized. More and more TTX will form stable combinations with ASP and GLU residues.

Figure 3b shows the calculated binding energies for ASP/TTX and GLU/TTX interactions. These results are consistent with the hydrogen bond data. Because TTX does not dock onto GLN, the GLN-TTX binding energy is considered to be zero. Furthermore, GLU exhibits a lower binding energy to TTX than ASP. Both the hydrogen bond analysis and the binding energy calculations indicate that ASP and GLU bind TTX more strongly than GLN does. Thus, mutating GLU to GLN significantly reduces the peptide’s ability to bind TTX. This diminished capacity of the nanostructure to bind TTX matches experimental observations in which the mutated nanostructure loses its sustained-release effect [19].

It is crucial to replicate these experimental observations, which show that TTX binds more tightly to ASP and GLU than to GLN. However, it is of particular interest to understand the specific interaction patterns that give rise to these differences, as such details are very challenging to obtain experimentally. Although GLN and GLU are both structurally similar and hydrophilic, their binding affinities to TTX differ markedly. It is essential to understand the fundamental reasons for this discrepancy. We hypothesize that the nanostructure’s binding to TTX requires interactions between the negatively charged carboxyl oxygen atoms of the nanostructure and the positively charged nitrogen atoms of TTX. To test this hypothesis, we used the radial distribution function (RDF) to analyze the distribution of TTX’s positively charged nitrogen atoms and nitrogen-bound hydrogen atoms near the nanostructure’s carboxyl oxygen atoms. Figure 3c shows the RDF results, averaged over the last 100 ns of ten trajectories. These results reveal that a large number of TTX hydrogen and nitrogen atoms are located at approximately 1.6 Å and 2.6 Å from the carboxyl oxygen atoms of GLU and ASP. Representative ASP-TTX and GLU-TTX interaction patterns are illustrated in Figure 3d,e. The carboxyl oxygen atoms of ASP and GLU serve as hydrogen bond acceptors, forming two parallel hydrogen bonds with the N–H groups of TTX. The distance between the carboxyl oxygen and the TTX hydrogen is about 1.6 Å, and the N–H bond length is about 1.0 Å, summing to a total distance of roughly 2.6 Å between the carboxyl oxygen of the nanostructure and the nitrogen of TTX. The RDF calculations are consistent with these representative interaction patterns. The sharp RDF peaks indicate that the interaction patterns observed in the simulations closely resemble the idealized configurations shown in Figure 3d,e. Beyond clarifying why GLN cannot form two hydrogen bonds with the nanostructure in the same manner as GLU and ASP, the representative interaction patterns also demonstrate that the amino group of GLN is sterically hindered from interacting with TTX as effectively.

### 2.3. TTX-Loading Capacity of the Peptides

Though the nanostructure reported from prior experiments is a significant achievement, one of the limitations is the loading capacity. One of the assumptions made here is that each GLU and ASP can bind one TTX molecule. With proper engineering of the peptide sequences, it is possible to engineer a second binding site to the peptide by just extending it by a few residues. To evaluate the TTX-sustained-release ability of different peptide sequences that stably form helical secondary structures, the loading number of TTX was defined. The loading number of TTX is equal to the number of TTX bound to ASP and GLU divided by the number of peptide chains, which is the average number of TTX stably bound to each peptide chain. The formula is shown in (1).

(1) $$Loading\;Number=\frac{N_{TTX\;bound\;to\;ASP\;and\;GLU}}{N_{peptide\;chains}}$$

**Figure 4:**
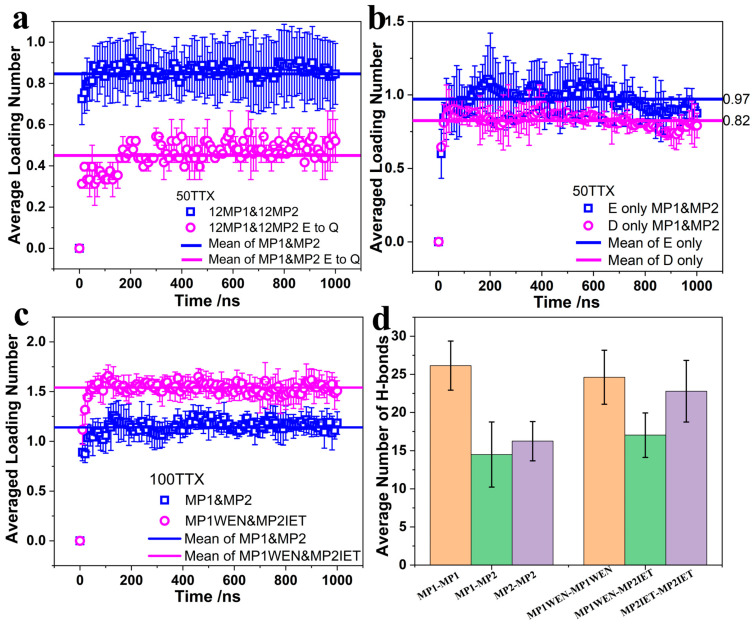
(**a**) Loading number of the system 12MP1&MP2 and the system 12MP1&MP2 (E to Q) with an error bar. The results of the 12MP1&MP2 system are the average of 10 trajectories. The result of 12MP1&MP2 (E to Q) is the average of 2 trajectories. (**b**) Loading number of the system 12MP1&MP2 (E only) and the system 12MP1&MP2 (D only) with an error bar. The presented results are the average of 2 simulation trajectories. (**c**) Loading number of the system 12MP1&MP2 and the system 12MP1WEN&MP2IET with an error bar. Add 100 TTX to the simulation box to make the binding of peptides to TTX as saturated as possible. The presented results are the average of the 5 simulation trajectories. (**d**) Number of hydrogen bonds between peptides with error bars. When calculating the hydrogen bonds between peptides of the same type (MP1-MP1, MP2-MP2, MP1WEN-MP1WEN and MP2IET-MP2IET), intramolecular hydrogen bonds were excluded. Brown represents the interaction between MP1 and MP1-like peptide chains. Green represents the interaction between MP1 and MP1-like peptide chains and MP2 and MP2-like peptide chains. Purple represents the interaction between MP2 and MP2-like peptide chains.

Ji et al. showed that mutating GLU (E) in the peptide sequence to GLN (Q) significantly weakens its binding ability and TTX sustained release ability [19]. As shown in our simulations, this weakening of TTX binding effects is well reflected by the loading number. As shown in Figure 4a, the average loading numbers of the two systems, MP1&MP2 and MP1&MP2 with an E-to-Q mutation, were calculated. The results displayed are the average of all trajectories. It can be seen that the loading number of the MP1&MP2 system is only about 0.9. This indicates that during the self-assembly interaction process of the peptide chain, ASP and GLU are nearly half occupied. When one GLU (E) of each peptide chain is mutated to GLN (Q), the number of amino acids that can bind TTX molecules on the peptide chain is reduced to half of the original number. The greatly reduced loading number at this time is shown as the blue curve in Figure 4a. This may be because the TTX loading capacity of the peptide chains with an E-to-Q mutation is significantly weakened and TTX bind only to ASP, which is consistent with the experimental results. At the same time, the loading number was used to further compare the differences between ASP (D) and GLU (E) in terms of their ability to bind TTX. MP1&MP2 peptide sequences containing only ASP (D) or only GLU (E) were designed, and the corresponding molecular dynamics simulations were carried out. The average loading number of E-only-MP1&MP2 and D-only-MP1&MP2 systems (Figure 4b) illustrates the different abilities of ASP and GLU to bind TTX. The average loading number of the last 100 ns of the E-only-MP1&MP2 system is 0.98, while that of the D-only-MP1&MP2 system was only 0.87. This calculation is consistent with previous calculations of binding energy and hydrogen bond number. Both ASP and GLU have a stronger ability to bind TTX. Although ASP and GLU have very similar structures, they have slightly different abilities to bind TTX. When there is ample space, there is no significant difference in the number of hydrogen bonds between ASP-TTX and GLU-TTX (Figure S3). We propose that GLU, having a longer side chain than ASP, reduces the effect of steric hindrance on TTX binding.

Although understanding how TTX binds to GLU and ASP is important, the critical problem we aimed to address is how to increase the loading capacity by adding more ASP and/or GLU residues to the designed peptides. To achieve this, two simulation systems were established for comparison. In one system, peptides MP1 and MP2 were placed in simulation boxes with 100 TTX molecules. In the other system, newly designed peptide sequences, MP1WEN and MP2IET, were used alongside 100 TTX molecules. MP1WEN and MP2IET were designed by adding three additional amino acids, including one GLU, to the original MP1 and MP2 sequences. The inclusion of only one GLU among the three additional residues was due to spatial exclusion between different TTX-binding residues. The loading numbers of the MP1&MP2 system and the MP1WEN&MP2IET system are shown in Figure 4c. In the presence of excess TTX, the loading number of the MP1&MP2 system is approximately 1.1, while that of the MP1WEN&MP2IET system exceeds 1.5. It is important to note that the addition of one GLU residue was expected to increase the loading number by 0.5, as calculated previously. The final loading number of 1.5 aligns well with our initial calculations. To ensure that the design and modification of the peptide sequences retain their ability to self-assemble and form nanostructures, secondary structure analysis (Figure 2c) and hydrogen bond calculations (Figure 4d) were performed to study the interactions between peptides. In the MP1WEN&MP2IET system, the proportion of helices was higher than in the MP1&MP2 system. This increased proportion of helices indicates that adding additional binding sites does not compromise the secondary structure stability of the peptide sequences MP1WEN and MP2IET. The stable helical secondary structure enhances the amphipathic properties of the peptides, thereby improving the self-assembly process. This observation is supported by the calculated number of hydrogen bonds between peptides. The results show that adding additional binding sites does not disrupt interactions between peptide chains, suggesting that the self-assembly ability of the peptides remains unaffected. In terms of the average number of hydrogen bonds, the interactions between MP2IET and MP1WEN are stronger than those between MP2 and MP1. We infer that MP1WEN and MP2IET exhibit superior self-assembly capabilities compared to MP1 and MP2.

## 3. Discussion

Despite advancements in understanding protein-related biochemical processes through theoretical methods, the application of computational techniques to explore the self-assembly of peptide chains and the formation of nanostructures remains underdeveloped. Peptide systems exhibit greater degrees of freedom which complicates simulations [35,36]. It is important to note that the self-assembly of peptides does not require a specific secondary structure. Rather, the self-assembly of peptides requires each molecule to possess a relatively uniform and regular structure. Our results suggest significant differences in the secondary structures of MP1 and MP2, which incorporate hydrophobic amino acid sequences, as compared to P1 and P2. Notably, MP1 and MP2 predominantly form stable α-helical structures, while P1 and P2 primarily exist as random coils, as supported by the ColabFold results and MD simulations. The stable α-helical structure is crucial for the self-assembly of MP1 and MP2 into TTX-sustained-release delivery nanostructures.

Previous studies by Wang et al. [37] and Fung Shan-Yu et al. [38] have also suggested the potential for peptide self-assembly via β-sheets. However, this type of assembly imposes stricter requirements for the uniformity and regularity of peptide sequences and interaction patterns, which primarily results in the formation of microtubules. Our findings substantiate the potential of theoretical methods to assist the research and design of peptide-based TTX carriers. Nonetheless, these results should be interpreted cautiously, considering several limitations. The time and spatial scales achievable by current all-atom molecular dynamics simulations often do not align with experimental conditions which makes fully converged nanostructure simulations challenging. However, through nuanced insights, theoretical methods offer valuable speculation on the local states of peptides, facilitating further improvement and optimization of peptide-based TTX carriers.

Research by Ji et al. [19] and several Cryo-EM experiments on sodium ion channel proteins [30,31,32,33,34] have established that ASP and GLU are critical binding sites for TTX. Our data indicate that ASP and GLU exhibit a significantly stronger binding affinity for TTX than GLN. Notably, despite their similar structure, GLU binds TTX more robustly than ASP. Additionally, we demonstrate ideal representative interaction patterns for ASP and GLU with TTX. Namely, the two carboxyl oxygen atoms of these residues are arranged in a V-shape and serve as dual hydrogen bond acceptors. This allows ASP and GLU to simultaneously form two or even three hydrogen bonds with the positively charged nitrogen terminal of TTX. This interaction pattern is relevant for research aiming to explore the mechanisms of other neurotoxins with structures similar to TTX. Furthermore, these interaction patterns can assist researchers in rapidly screening other potential molecules that could function as TTX binding carriers. Potential TTX binding molecules with similar V-shaped acceptor structures can be explored. While previous studies have focused on protein interactions with TTX, our findings suggest that a broader range of organic molecules, such as uracil, may serve as potential TTX carrier molecules. Moreover, the structural similarities between TTX and STX, particularly in their functional regions, indicate that insights derived from TTX research extend to STX. Researchers can develop carriers targeting both neurotoxins (TTX and STX) while gaining a better understanding of STX, which has greater potential than TTX as a local anesthetic.

Based on the simulation results for MP1, MP2, and their E-to-Q mutant variants, we propose that the TTX binding capacity of peptides correlates directly with the number of GLU and ASP residues. Given this hypothesis, we designed MP1WEN and MP2IET, which enhance TTX binding sites by increasing GLU content. Our data indicate that MP1WEN and MP2IET not only exhibit superior binding and loading capacities for TTX, but they also maintain a stable α-helical structure, similar to the peptide–peptide interaction levels observed in MP1 and MP2. The application of theoretical approaches in protein and peptide research is demonstrated by our study. Specifically, we assert that utilizing theoretical methods to refine and design peptide-based TTX delivery nanostructures is both viable and beneficial.

However, a few limitations of current work must be discussed. First, from a methodological standpoint, examining multiple protein structure prediction tools other than AlphaFold 2/ColabFold, such as RosettaFold All-Atom and ESMFold, can enrich our understanding of how sensitive the predicted structure ensemble is to a model’s underlying algorithmic assumptions. Using more than one ML-based predictor often provides complementary insights into structural variability, increasing our confidence in regions consistently predicted across models and highlighting regions of uncertainty where predictions differ. On the downside, each model has its own training biases, performance characteristics, and resource demands, so pursuing multiple predictions from different tools can be computationally expensive, time-consuming, and may not necessarily converge toward a single consensus structure. In addition, integrating and rationalizing discrepancies among predictions can prove non-trivial, requiring additional expertise and analysis to produce a coherent interpretation of the structural ensembles. Furthermore, while we have taken steps to mitigate single-trajectory biases by averaging over multiple independently initiated simulations, it remains challenging to guarantee that the full spectrum of relevant kinetic events has been adequately sampled within the accessible simulation timescales. Although the peptide systems studied here are more flexible and dynamic than rigid protein assemblies, potentially offering a broader range of conformational states, fully mapping their complex free-energy landscapes is inherently difficult. The absence of the direct analyses of kinetic parameters—for instance, through self-diffusion coefficients or autocorrelation functions of center-of-mass trajectories—means that we cannot definitively confirm ergodic sampling or complete equilibration. Furthermore, technical and computational constraints prevent us from simulating the entire trajectory of complex self-assembly processes. As a result, our conclusions must be interpreted with the understanding that the underlying timescales and mechanistic pathways associated with transitions between structural states, as well as large-scale aggregation events, may not be fully resolved by the current simulations. When defining the loading number calculation method, we were unable to account for hydrogen bond occupancy. Nonetheless, our small-scale simulations indicate that GLU-TTX and ASP-TTX hydrogen bonds can persist for more than 1 ns. Additionally, in classical force field molecular dynamics simulations, interaction assessments are largely based on distance criteria, raising concerns about the accuracy and reliability of such evaluations.

Despite these limitations, given the current computational resources and methodological frameworks, MD simulations remain a powerful tool for guiding the design of peptide-based, TTX-sustained-release delivery systems. Moving forward, research efforts should focus on developing simulation techniques capable of accurately modeling peptide self-assembly across relevant time and length scales. Such advancements will enable a more comprehensive investigation into the mechanisms underlying TTX-sustained release.

## 4. Methods and Materials

The initial peptide structures used for the molecular dynamics (MD) simulations were generated using ColabFold [27], as shown in Figure 1. After generating the peptide structures, force field parameters for all-atom MD simulations were obtained using the PDB Reader and Manipulator module of the CHARMM-GUI web server [39,40,41]. For tetrodotoxin (TTX), the initial structure and corresponding force field parameters were prepared using the Ligand Reader and Modeler module of CHARMM-GUI [42,43]. The initial simulation boxes were constructed using the Multicomponent Assembler module of CHARMM-GUI, as depicted in Figure 1b. Peptides and TTX molecules were randomly placed inside the simulation box, along with potassium and chloride ions to maintain electroneutrality and TIP3P water molecules for solvation [44]. Notably, all peptides were modeled using their ColabFold-generated structures with the highest pLDDT confidence scores, ensuring that their initial conformations were as reliable as possible. No structural constraints were applied to the peptides during the production runs, allowing them to undergo unbiased conformational changes over time. Such unconstrained sampling methods have been widely used to enhance conformational diversity and improve our understanding of peptide-ligand interactions. A total of eleven simulation systems were established, varying in the number of peptide chains, the number of TTX molecules, simulation lengths, and the number of trajectories, as summarized in Table 1. The CHARMM36m force field was used in all simulations, known for its refined treatment of peptide backbone and side-chain conformations [45], was employed for all simulations. A 10 × 10 × 10 nm^3^ solvation box was used to accommodate peptide nanostructures and allow for sufficient space for possible peptide rearrangements. In cases where larger systems were required, a 15 × 15 × 15 nm^3^ box was utilized. All systems were neutralized by adding counter ions.

Energy minimization was performed using GROMACS2023.1 [46,47] for a maximum of 5000 steps, targeting a force tolerance of 1000.0 kJ/mol/nm to remove non-physical contacts. Subsequently, a 1 ns (1,000,000 steps) NPT ensemble simulation was executed to equilibrate the systems. The LINCS algorithm [48] was employed to constrain bond lengths involving hydrogen atoms, while the temperature was maintained at 303 K using the Nose–Hoover thermostat [49]. The pressure was kept at 1 atm via the Parrinello–Rahman barostat. Periodic boundary conditions were applied in all three Cartesian dimensions. Long-range electrostatics were computed using the Particle Mesh Ewald (PME) method [50,51,52], and both van der Waals and Coulomb interactions were truncated at a cutoff distance of 12 Å. Production simulations were carried out for 1000 ns to ensure adequate sampling. Secondary structural analyses of the peptide trajectories were performed using do_dssp, which applies the DSSP algorithm [53] for backbone hydrogen bonding pattern analysis. Visualization and trajectory analysis were conducted using VMD and Chimera [54,55].

For binding free-energy calculation systems, initial simulation systems, each consisting of a peptide and a TTX molecule randomly placed in a 5 × 5 × 5 nm^3^ simulation box, were constructed using CHARMM-GUI. Potassium and chloride ions, as well as water molecules, were included to ensure proper solvation and system neutrality. During the 1000 ns simulations, the peptide–TTX complexes formed rapidly and remained stable, reflecting the strong binding tendency of the designed peptide sequences. After the completion of the MD simulations, the final conformations were extracted for subsequent docking procedures. For the binding free energy calculations of peptide–TTX interactions, we employed the Molecular Operating Environment (MOE 2022.02) platform [37], which has been widely utilized for protein–ligand docking and scoring. Four specifically designed peptides (FFFLLGTQAYWEN, FFFLLGCGAWIDT, FFFLLGCGAWIQT, FFFLLGCGAWIET), each containing one aspartate (ASP) and one glutamate (GLU) residue, were simulated in the presence of a single TTX molecule. These acidic residues are known to form strong interactions with cationic groups, potentially enhancing binding affinity to TTX/STX [56]. For docking, we employed the Site Finder and molecular redocking functionalities within MOE’s General Dock panel. Both the “Placement” and “Placement Score” options were set to “None” to maintain consistency with the predicted structures obtained from the simulations. In the refinement step, the peptides were treated as rigid to preserve their experimentally or computationally predicted conformations, whereas TTX was considered in both rigid and flexible forms to evaluate the impact of ligand flexibility on binding. The GBVI/WSA ΔG scoring function was used to estimate the binding free energies of the peptide–TTX complexes, providing a quantitative measure of their predicted binding affinities. This integrated approach, combining advanced structure prediction, rigorous MD simulations, and careful free energy calculations, builds on established computational methodologies and previous studies that have utilized similar protocols to understand peptide–ligand interactions in complex biological systems. The results generated from these simulations offer valuable insights into the structural determinants of peptide–TTX binding and inform future experimental validation and rational peptide design strategies.

## 5. Conclusions

In this article, we conducted a series of simulations on peptide-based nanoparticle TTX-sustained-release carrier systems, demonstrating the effectiveness of using computational chemistry methods to aid in their design and enhancement. Through peptide predictions made with ColabFold2, we found that specific sequence orders and compositions (such as MP1 and MP2) enable peptides to form stable helical secondary structures. By comparing the consistencies and discrepancies between experimental and simulation results, we observed that only peptides with stable helical secondary structures can create transport carriers that exhibit favorable binding and sustained release capabilities for TTX. While our results may not constitute definitive evidence, they provide valuable insights into the underlying mechanisms of the aforementioned carrier systems. The self-assembly of nanoparticles requires a composition of structurally uniform amphiphilic peptides, which is precisely what helical secondary structures offer. In contrast, peptides characterized by random coils introduce excessive degrees of freedom which leads to varied self-assembly outcomes. Furthermore, we employed molecular dynamics to examine the differences in the binding affinities of various amino acids for TTX. These analyses corroborate the experimental conclusions regarding TTX’s mechanism of action on sodium channel proteins, specifically highlighting TTX’s strong binding affinity for ASP and GLU residues near the sodium ion channel gate. This binding is notably robust and can persist over extended periods, significantly disrupting channel protein function. Our simulations show how computational chemistry methods can effectively distinguish the binding abilities of different amino acids to TTX. These methods can aid the design and optimization of peptide-based nanoparticle TTX-sustained-release carriers.

Additionally, we defined a loading number function to characterize the differences in binding and TTX-carrying capabilities among various peptides. Our findings indicated that reducing the number of ASP and GLU residues diminishes the peptides’ TTX-carrying capacity, which led us to hypothesize that increasing these residues would enhance binding capacity. We then designed and simulated peptides with additional binding sites. Increasing the binding sites correlated with a greater incorporation of excess TTX, confirming that the peptides’ ability to bind and carry TTX was enhanced. While our current simulations represent a preliminary application of computational chemistry methods to nanoparticle design and enhancement, they serve as a solid foundation for future research. As we deepen our understanding of peptide-based nanoparticle TTX-sustained-release carriers, we also aim to improve the peptide design process. Overall, the integration of experimental and computational chemistry approaches will facilitate more efficient research on and development of high-performance TTX-sustained-release carriers.

## Figures and Tables

**Table 1:** System settings of the simulations.

System		Number of Peptides	Number of TTX	Time and No. Trajectory	Number of Atoms	Size of Box
1	P1&P2+TTX	12P1+12P2	50	1000 ns × 10	92,136	10 × 10 × 10 nm^3^
2	MP1&MP2+TTX 1	12MP1+12MP2	50	1000 ns × 10	91,789	10 × 10 × 10 nm^3^
3	MP1&MP2+TTX 2	24MP1+24MP2	50	1000 ns × 2	89,999	10 × 10 × 10 nm^3^
4	MP1&MP2+TTX 3	12MP1+12MP2	100	1000 ns × 5	90,943	10 × 10 × 10 nm^3^
5	MP1WEN&MP2IET+TTX	12MP1WEN+12MP2IET	100	1000 ns × 5	316,541	15 × 15 × 15 nm^3^
6	MP1&MP2+TTX (E to Q)	12MP1+12MP2 (Mutated E to Q)	50	1000 ns × 2	92,246	10 × 10 × 10 nm^3^
7	MP1&MP2+TTX (E to D)	12MP1+12MP2 (Mutated E to D)	50	1000 ns × 2	92,074	10 × 10 × 10 nm^3^
8	ScMP1&ScMP2+TTX	12ScMP1+12ScMP2	50	1000 ns × 2	91,418	10 × 10 × 10 nm^3^
9	MP1&MP2+TTX (E&D to Q)	12MP1+12MP2 (Mutated E&D to Q)	50	1000 ns × 2	92,263	10 × 10 × 10 nm^3^
10	MP1&MP2+TTX (E only)	12MP1+12MP2 (Mutated D to E)	50	1000 ns × 2	91,693	10 × 10 × 10 nm^3^
11	MP1&MP2+TTX (D only)	12MP1+12MP2 (Mutated E to D)	50	1000 ns × 2	91,810	10 × 10 × 10 nm^3^

## Data Availability

The data supporting the findings of this article are available upon email request to the corresponding author.

## Supplementary Materials

The following supporting information can be downloaded at: https://www.mdpi.com/article/10.3390/molecules30010061/s1, Figure S1: Predicted structure from Colabfold2, sequence of peptides, and predicted IDDT of MP1EtoD, MP2EtoD, MP1EDtoQ, MP2EDtoQ, MP1Eonly, MP2Eonly, MP1Donly, MP2Donly, MP1EtoQ, and MP2EtoQ.; Figure S2: Sequence and predicted IDDT of P1, P2, MP1, MP2, ScMP1, ScMP2, MP1WEN, and MP2IET. Figure S3: (a) Hydrogen bond binding between MP2_1Eonly and TTX. The peptides only contain 1 GLU. Another GLU was mutated to ALA. The hydrogen bonds are shown in pink. (b) Number of hydrogen bonds between GLU and TTX during 1000 ps simulation. (c) Hydrogen bond binding between MP2_1Donly and TTX. The peptides only contain 1 ASP. Another GLU was mutated to ALA. The hydrogen bonds are shown in pink. (d) Number of hydrogen bonds between ASP and TTX during 1000 ps simulation.
