# Supplementary material for: Molecular Dynamics Insights into Peptide-Based Tetrodotoxin Delivery Nanostructures
Source: Molecules. 2024 Dec 27;30(1):61. doi: 10.3390/molecules30010061 (PMC11721190; doi:10.3390/molecules30010061)
Supplement: Supplementary file 1 [file molecules-30-00061-s001.zip › molecules-3342709-supplementary.pdf]

SUPPLEMENTARY MATERIALS

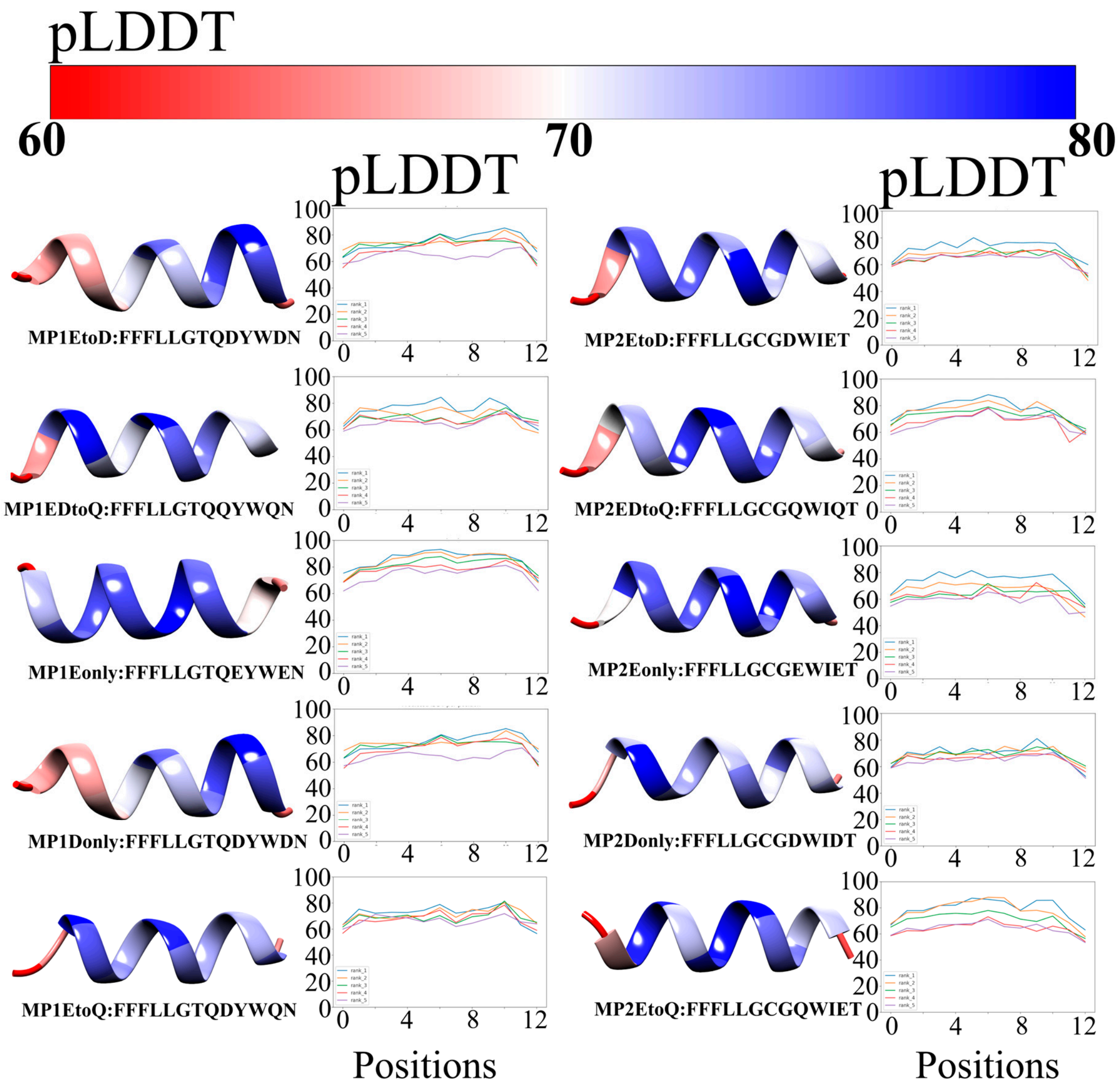

Figure S1. Predicted structure from Colabfold2, sequence of peptides and predicted lDDT of MP1EtoD, MP2EtoD, MP1EDtoQ, MP2EDtoQ, MP1Eonly, MP2Eonly, MP1Donly, MP2Donly, MP1EtoQ and MP2EtoQ. Residues are colored according to their pLDDT.

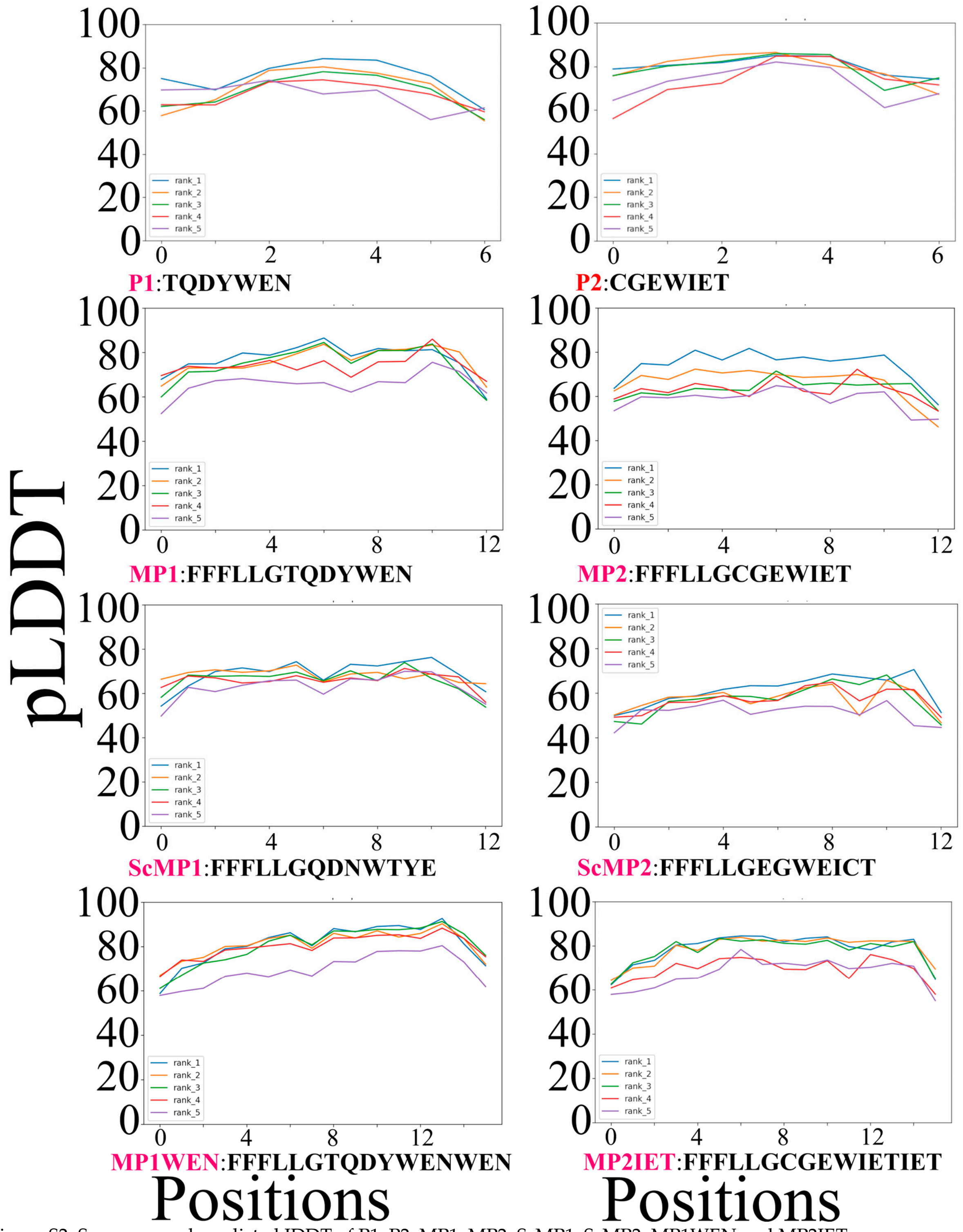

Figure S2. Sequence and predicted lDDT of P1, P2, MP1, MP2, ScMP1, ScMP2, MP1WEN and MP2IET.

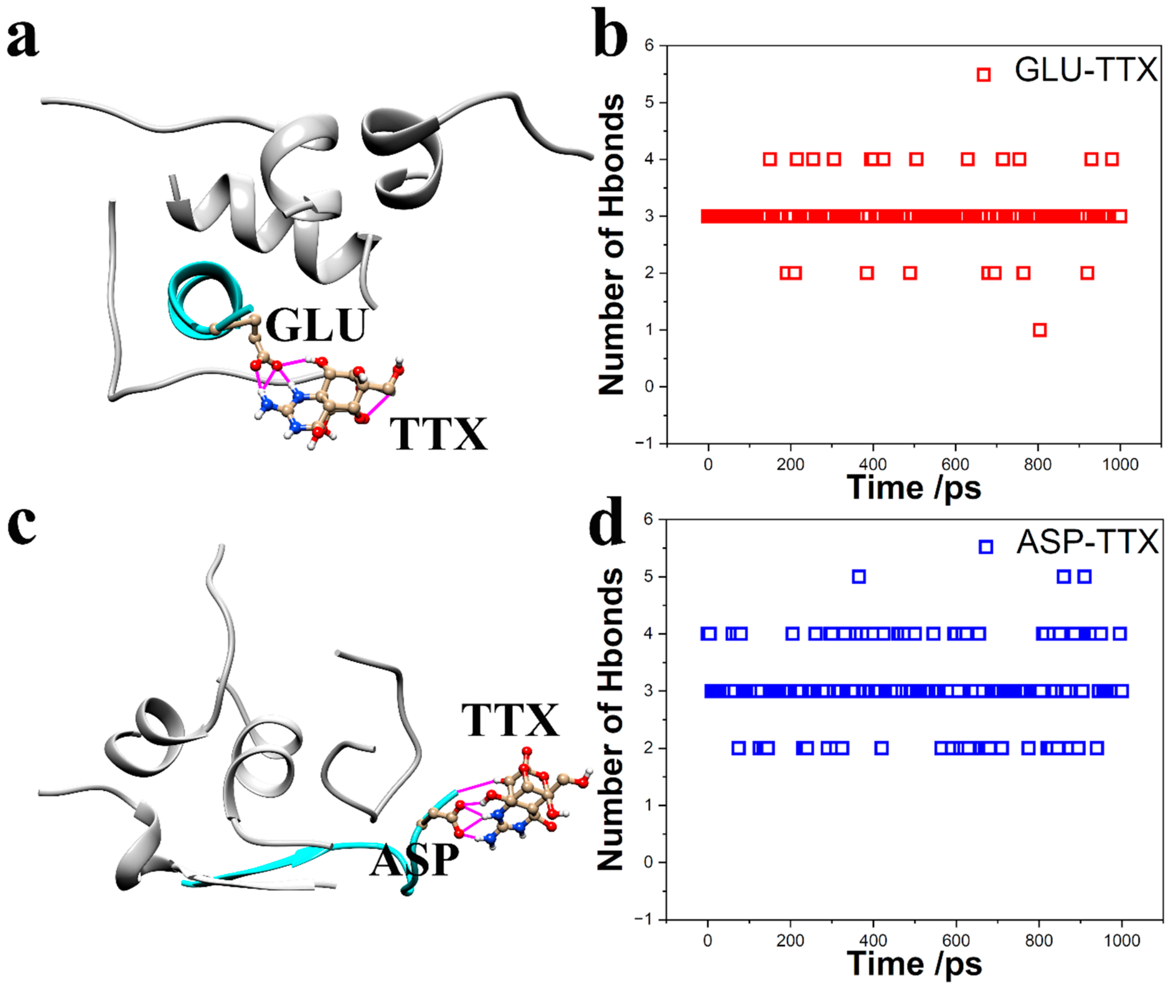

Figure S3. (a). Hydrogen bonds binding between MP2_1Eonly and TTX. The peptides only contain 1 GLU. Another GLU was mutated to ALA. The hydrogen bonds were shown in pink. (b). Number of Hydrogen bonds between GLU and TTX during 1000ps simulation. (c). Hydrogen bonds binding between MP2_1Donly and TTX. The peptides only contain 1 ASP. Another GLU was mutated to ALA. The hydrogen bonds were shown in pink. (d). Number of Hydrogen bonds between ASP and TTX during 1000ps simulation.
